# Efficacy and safety of apatinib in advance osteosarcoma with pulmonary metastases: A single-center observational study

**DOI:** 10.1097/MD.0000000000011734

**Published:** 2018-08-03

**Authors:** Kai Zheng, Ming Xu, Lei Wang, Xiuchun Yu

**Affiliations:** Orthopedic Department, The General Hospital of Jinan Military Commanding Region, Jinan, China.

**Keywords:** apatinib, efficacy, osteosarcoma, safety, targeted therapy

## Abstract

This study was designed to evaluate the efficacy and safety of apatinib in patients with advanced osteosarcoma and pulmonary metastases following failed first-line chemotherapy.

There were 10 patients with osteosarcoma pulmonary metastases, whose first-line chemotherapy had failed, had received apatinib treatment as a single agent. All patients had at least 1 measurable lung tumor. Progression free survival (PFS), overall survival (OS), objective response rate (ORR), disease control rate (DCR), and treatment-related adverse effects (AEs) were reviewed and evaluated. Tumor response was assessed by response evaluation criteria in solid tumor criteria (RECIST). The 10 patients in this study received apatinib treatment for 2 to 16 months with a median of 7.5 months. The median PFS was 7.5 months. The 6-month, 8-month, and 10-month PFS rates were 60%, 40% and 26.6%, respectively. The median OS was 14 months. After 6-month apatinib treatment, 2 patients achieved partial response and 5 patients achieved stable disease, while 3 patients were evaluated as progression of the disease. At the 6-month follow-up, the ORR was 20.0% and the DCR was 70.0%. Hand-foot syndrome with grade 1/2 was the most common treatment-related AE. No drug-related severe AEs occurred.

After failed first-line chemotherapy, apatinib as a single agent exhibited efficacy and acceptable safety in patients with advanced osteosarcoma and pulmonary metastases.

## 1 Introduction

Osteosarcoma is the most common primary high-grade sarcoma of the human skeleton which originates from mesenchymal tissue. In the United States in 2017, the estimated number of new bone sarcoma was 3260 and about 1550 deaths from this disease were expected.^[1]^ The National Central Cancer Registry of China reported that the estimated number of new bone sarcoma in China in 2015 was 28,000 and the estimated number of death due to bone sarcoma was 20,700.^[2]^ The treatment for osteosarcoma is not easy, and death due to osteosarcoma relapse and metastasis is common in United States and even more common in China. Despite combined chemotherapy and surgery, approximately one-third of the patients still relapse, and three-fourths of patients develop metastases.^[3,4]^ It has been reported that about 90% of osteosarcoma relapses are due to pulmonary metastases.^[5]^

The first-line treatments for osteosarcoma are recommended in the National Comprehensive Cancer Network (NCCN) guideline. The classical chemotherapy drugs for osteosarcoma include methotrexate, cisplatin, ifosfamide, and doxorubicine.^[6]^ However, the treatment for patients following failed first-line chemotherapy is controversial.^[7]^ Some authors have reported that the overall survival (OS) rate of patients with advanced osteosarcoma is 0% to 50%.^[8]^ Therefore, efforts should be made to discover a new therapy for the treatment of advanced osteosarcomas.

Persistent angiogenesis is a typical feature of malignant tumors which plays an important role in tumor growth, invasion and metastasis. Thus, targeting tumor angiogenesis is considered an important antitumor therapy.^[9]^ Apatinib, a small molecule tyrosine kinase inhibitor with a high selectivity for vascular Endothelial growth factor receptor 2 (VEGFR2), exerts promising antitumor effect in various tumors.^[10]^ In a third-phase clinical trial, apatinib has been proven to be a safe and effective drug in patients with advanced gastric cancer.^[11]^ On that basis, apatinib was granted approval for the treatment of advanced gastric cancer by the China Food and Drug Administration (CFDA) in 2014. In addition, apatinib has also been reported to be successful in the treatment of some advanced cancers.^[12–17]^ Several case reports about apatinib treatment of osteosarcoma and a special type of soft tissue sarcoma has been published.^[14,18,19]^ In this study, apatinib is used as a single agent to treat patients with advanced osteosarcoma and pulmonary metastases after failed first-line chemotherapy. The details and results of the treatments are presented and the efficacy and safety of apatinib are discussed.

## 2 Methods

### 2.1 Patients and patient's selection criteria

Ten patients with osteosarcoma received apatinib treatment at The General Hospital of Jinan Military Commanding Region from May 2016 to May, 2017, for at least more than 1 month. Patients with the following criteria were eligible for this study: pathological diagnosis of osteosarcoma was definite; patients had received first-line chemotherapy before apatinib treatment; measurable pulmonary metastases lesions; complete clinical, radiographic, and pathological records. Data extracted from the charts included sex, age, histology, treatment before apatinib, lung metastasis time, initial apatinib dose, medication time, and efficacy evaluation at the end of follow-up. All the patients were required to accept regular follow-up, which included physical and imaging examinations.

### 2.2 Treatment plan, drug dose adjustment

Patients received a daily dose of oral apatinib at 500 mg for adults and 250 mg for children in tablet form. A treatment cycle was defined as 1 month. In each cycle, dose reductions were allowed up to a daily dose level not lower than 250 mg. Dose recovery was permitted after drug tolerance/adverse effect. Application of apatinib was in accordance with the Declaration of Helsinki and this work was approved by the Ethics Committee of Jinan Military General Hospital. All patients volunteered to participate in this trial and signed written informed consent.

### 2.3 Follow-up schedule, tumor response assessment, and survival time

There were 2 primary end points: the OS, which was defined as the duration from the time of random assignment to the time of death, and PFS, assessed by investigators and verified by independent radiologists. Secondary end points included the objective response rate (ORR; including rate of complete response plus partial response), DCR (including complete response, partial response, and stable disease), and safety. Complete response (CR) means the tumor completely disappeared for more than 1 month. Partial response (PR) means the tumor was reduced by at least 30% for at least 4 weeks. Stable disease (SD) means the sum of the maximum diameter of the target lesion was reduced to less than the PR, or increased to less than the progression of the disease (PD). The PD means that the maximum diameter of the target lesion increases by at least 20%, or new lesions occur.

Tumor evaluations were performed at baseline, after cycles 2 and 3, and every 4 weeks thereafter until disease progression, which was based on CT scan analysis as defined by RECIST (version 1.1). Adverse events (classified and graded using the National Cancer Institute Common Terminology Criteria for Adverse Events [version 3.0]) were assessed at baseline (after patients provided written informed consent) until at least 28 days after the last dose of the study drug was administered.

### 2.4 Statistical analyses

The survival and safety analyses were performed for patients who received at least 1 month of apatinib. Life table and Kaplan–Meier survival curves were used for PFS estimation. Data analysis and curve plotting were performed using the GraphPad Prism 5.0 software (GraphPad Inc., La Jolla, CA).

## 3 Results

### 3.1 Patients and tumor characteristics

**Figure 1:**
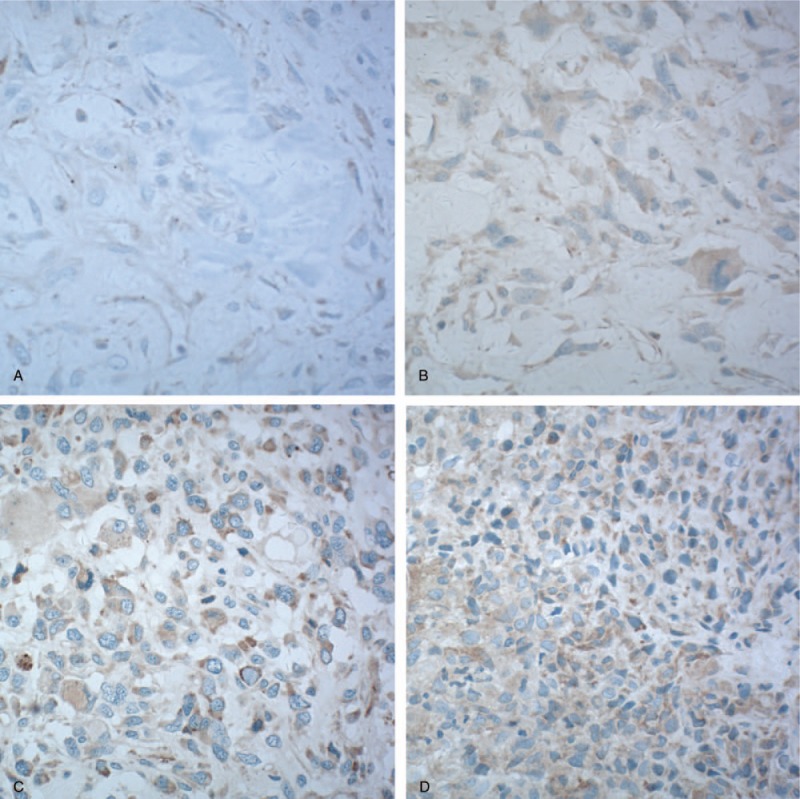
Immunochemical staining analysis of VEGFR2 in available tissue was performed before apatinib therapy. (A) Absence of expression of VEGFR2. (B) Weak expression of VEGFR2. (C and D) Moderate expression of VEGFR2. VEGFR-2 = vascular endothelia growth factor receptor-2.

Three of the 10 osteosarcoma patients who were treated with apatinib following failed first-line chemotherapy had pulmonary metastases in the first diagnosis, while the other 7 patients developed pulmonary metastases at different times, ranging from 9 to 36 months, after operation (Table 1). The median age was 16 years (range 12–30 years). All the patients had received prior first-line chemotherapy with the same drugs, namely cisplatin, doxorubicin, and ifosfamide according to the NCCN guideline. Before apatinib treatment, 9 patients received limb salvage surgery, including primary limb osteosarcoma wide resection and bone defect reconstruction. After pulmonary metastases were controlled by apatinib, 1 patient underwent hip disarticulation in order to get rid of a huge tumor load in the thigh. All patients were treated for more than 1 month with apatinib, and immunohistochemical staining analysis of VEGFR2 was performed in tissue from 5 of these patients whose tissue before apatinib therapy was available. Different expression was detected, but no consistent relationship was confirmed in this study (Fig. 1).

**Table 1:**
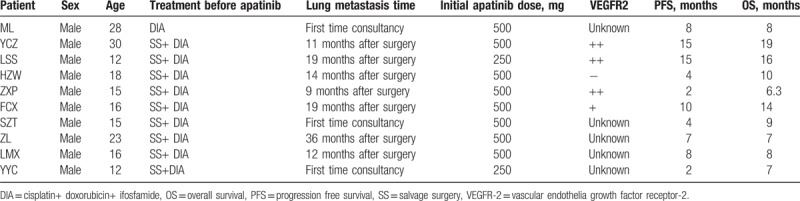
The clinical characteristics of 10 osteosarcoma patients with lung metastasis treated with apatinib.

### 3.2 Efficacy

**Figure 2:**
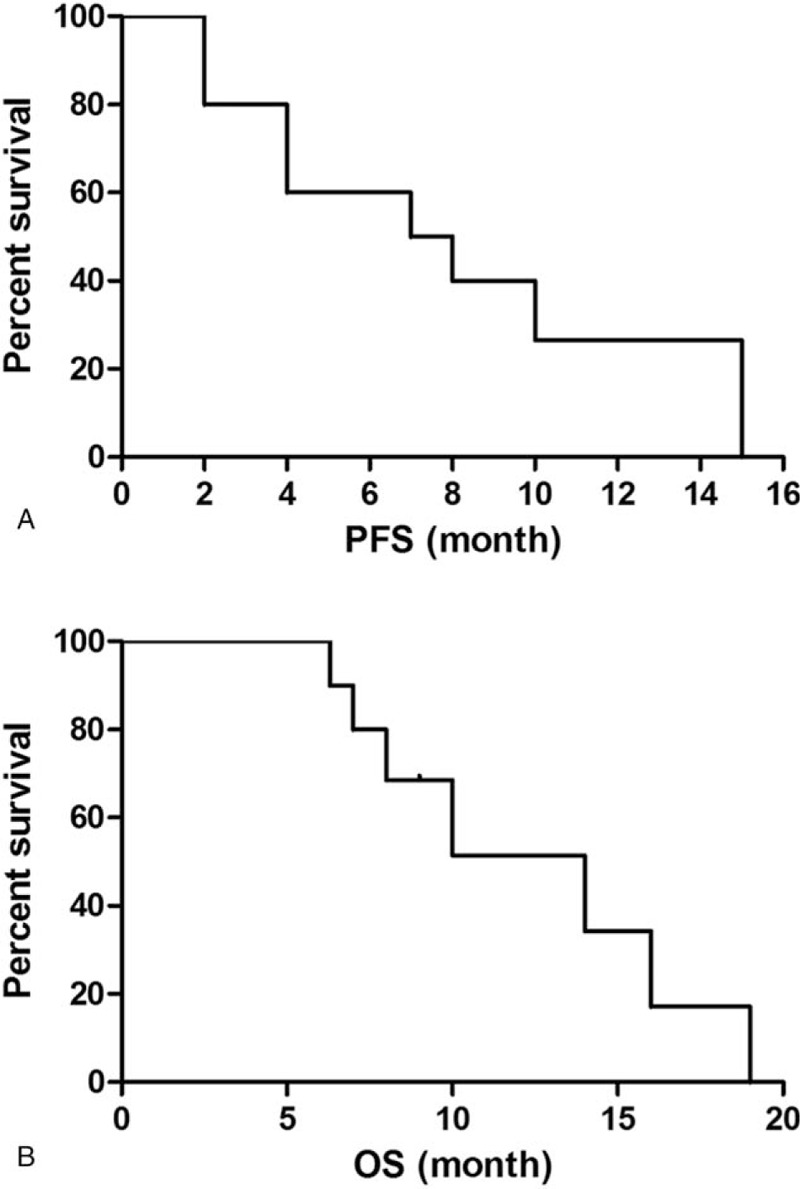
Efficacy evaluation of apatinib in patients with advance osteosarcoma and pulmonary metastases. (A) PFS curve in these patients. (B) OS curve in these patients. PFS = progression free survival.

**Figure 3:**
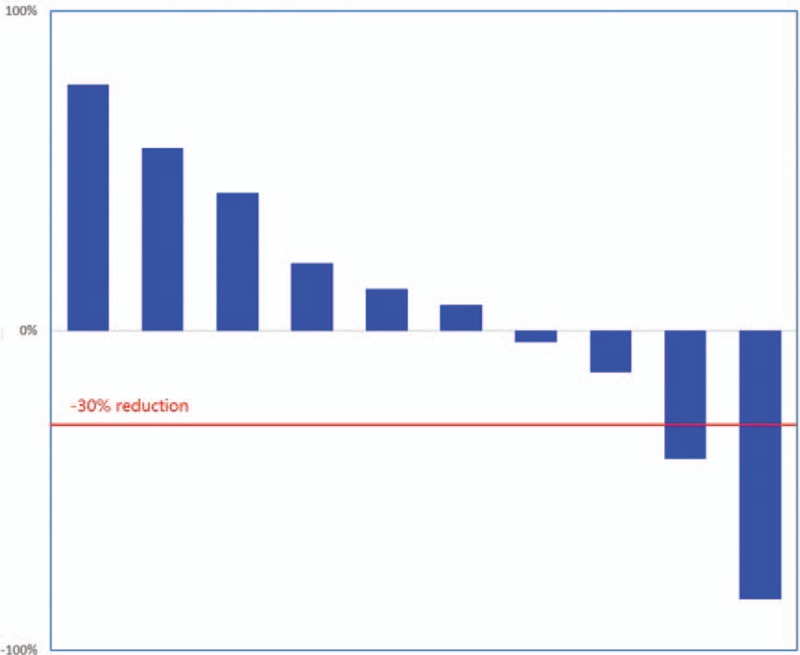
The tumor change of pulmonary metastases at 6 months after apatinib treatment.

The median follow-up for efficacy was 7.5 months. At the last follow-up, 2 patients (20%) remained on therapy at 8 and 7 months, respectively. Progression in 7 (70%) patients and accidental death in 1 (10%) patient were reasons for drug treatment interruption. Three (30%) patients received apatinib for >1 year. Six patients were free from progression after 6 months of therapy. The PFS rate was 60% at the 6-month visit, 40% at the 8-month visit, and 26.6% at the 10-month visit (Fig. 2A). The median OS was 14 months (Fig. 2B). After 6 months of apatinib treatment, we observed no complete responses (CRs), but 2 (20%) patients did meet the criteria for PR (Fig. 3). Five (50%) patients qualified for SD. The disease control rate (DCR) was 70% at the 6-month follow-up. Six patients died before the last follow-up due to disease progression. One patient died without lung tumor progression.

**Figure 4:**
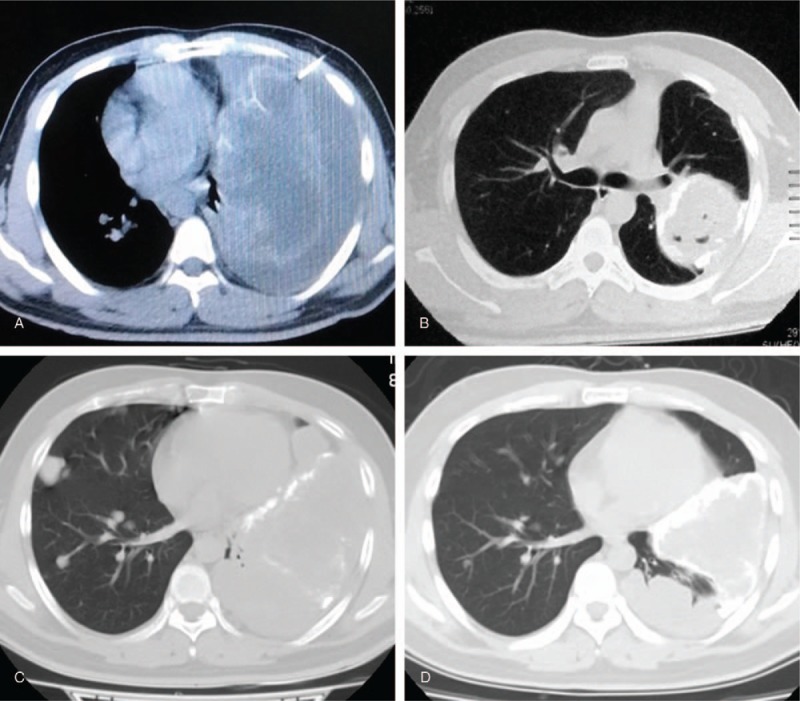
Image illustrating tumor changes of 1 PR patient with lung metastatic osteosarcoma. (A) The chest CT shows large pulmonary metastases in the left lung. (B) Two months later after apatinib treatment. (C) Interruption of apatinib treatment for 1 month. (D) The patient's apatinib treatment recovery, the tumor achieved PR again.

**Figure 5:**
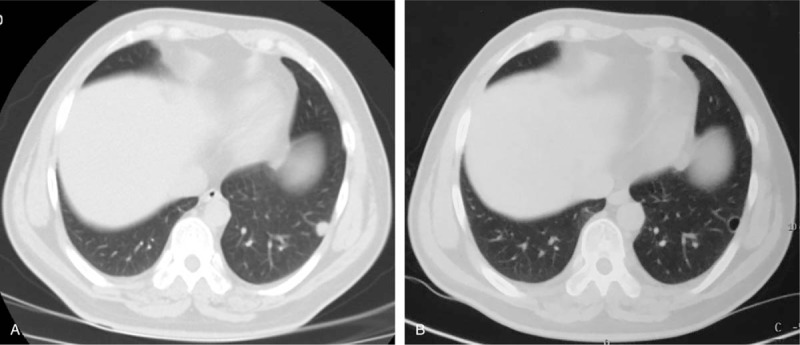
The chest CT shows the lesion replaced by a vacuole after apatinib treatment for 2 months.

Besides the detected pulmonary metastases after evaluation according to the Response Evaluation Criteria in Solid Tumors (RECIST) guidelines,^[20]^ other tumor changes were detected by imaging. In 2 patients, computed tomography (CT) scan of the lungs revealed that pleural effusion had completely disappeared, while marginal sclerosis and vacuoles inside the lesion were evident (Fig. 4). For 1 patient, after taking apatinib for 2 months, the CT scan images showed that the lesion was replaced by a vacuole (Fig. 5). Similar to other target drugs, interruption of the treatment with apatinib could lead to disease progression. Fortunately, the tumor in the lung of the patient showed PR again once the apatinib treatment was resumed after 1-month interruption (Fig. 4).

### 3.3 Adverse effects

The toxicity and side-effect events encountered in patients treated with apatinib are shown in Table 2. In general, the grade of severity of the drug-related adverse events was limited to grade 1 or 2. Overall, the grade 1 adverse reactions accounted for 54.5% of the total adverse events. Grade 2 and grade 3 adverse reactions accounted for 39.4 and 6.1%, respectively, but no grade 4 was observed in this study. We recorded at least 1 adverse event in all 10 patients. 31 (93.9%) of the 33 total adverse events were grade 1 to 2. The most common grade 1 to 2 adverse events were the following: hand-foot syndrome, 8 (80.0%) of 10 patients; cough, 4 (40.0%); fatigue, 3 (30.0%); hypertension, 3 (30.0%); pneumothorax, 3 (30.0%); weight loss, 3 (30.0%); diarrhea, 2 (20.0%); pain, 2 (20.0%); dizziness, 2 (20.0%), as listed in Table 2. All these adverse events were causally related to the study drugs. One (10.0%) patient who had a grade 3 hypertension received decrement and recovery drug dose in 1 month. We recorded no other serious adverse events during the treatment. No deaths were related to the apatinib treatment.

**Table 2:**
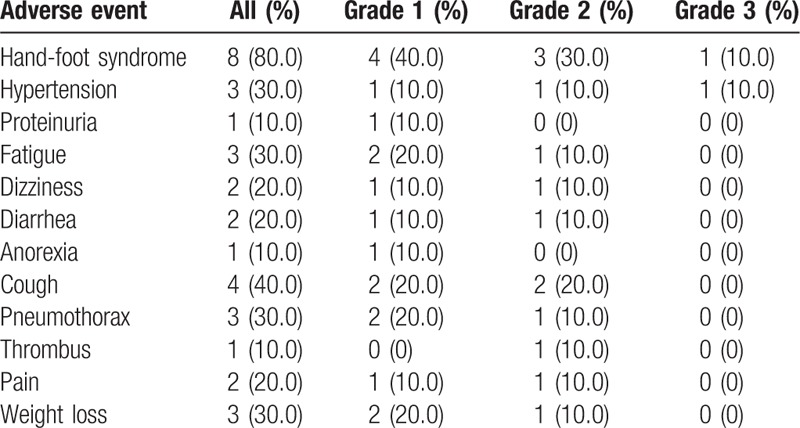
Adverse events that arose in at least 1 patient.

## 4 Discussion

Molecular targeted therapy plays an important role and has achieved success in the treatment of many refractory malignancies since imatinib was approved as a first-line drug to treat chronic myeloid leukemia by the Food and Drug Administration in 2001. Antiangiogenic targeted drugs are a large class of targeted drugs which achieve success in certain tumors treatments.^[21,22]^ At present, there is no effective drug for the targeted treatment of bone and soft tissue sarcoma. Apatinib is a novel and highly selective inhibitor of the VEGFR2 tyrosine kinase, which blocks the downstream signal transduction of VEGFR2 at the cellular level.^[23]^ Apatinib has been proven to be a safe and effective drug in patients with advanced gastric cancer. Also, some researchers have reported the successful treatment of osteosarcoma and other sarcomas.^[13,14,24]^ Nevertheless, in order to share our experience on the treatment of osteosarcoma patients with apatinib, this study describes the treatment of some patients and effects of apatinib in details.

Sorafenib, a multikinase inhibitor, has been recommended by the NCCN guidelines to treat unresectable and relapsed high-grade osteosarcoma as the second-line therapy based on the results of a phase II trial.^[25,26]^ A total of 35 patients were included in that multicenter phase II trial, and the results showed that the PFS at 4 months was 46% and the clinical benefit rate was 29%. Additionally, the median PFS and OS are 4 months and 7 months, respectively. In this study, apatinib is used to treat osteosarcoma following failed first-line chemotherapy. The results show that the PFS at 6 months was 60% and the DCR was 70.0%. In addition, the median PFS and OS were 7.5 months and 14 months, respectively. Admittedly, it is dogmatic to make a conclusion that the efficacy of apatinib is better than that of sorafenib. However, apatinib is a potential new option for the treatment of osteosarcoma with pulmonary metastases.

Noteworthy, tumor boundary sclerosis and tumor cavity formation are observed among PR and SD patients. We think that the tumor boundary sclerosis is a signal for tumor control and tissue repair. The formation of a tumor cavity is the manifestation of tumor ischemia and necrosis due to the interruption of tumor angiogenesis. Evaluation of the activity of tumor cells should be another evaluation criterion for tumor control, but not based only on the measurement of the tumor size.

Target detection is considered to be an important process before target drug treatment, while the number of target receptors is considered to be an important indicator of the efficacy of the targeted drugs. Apatinib, as a small molecule tyrosine kinase inhibitor, has a high selectivity for VEGFR2. In this pathway, targeted detection of VEGFR2 is performed in tumor specimens by immunohistochemical staining analysis. The results are not exactly as anticipated. Some patients with strong VEGFR2 expression did not benefit from the treatment with apatinib, whereas some patients with weak VEGFR2 expression were benefited by the treatment.

How to use apatinib in the treatment of patients with osteosarcoma is another question. In this study, all the patients received apatinib as a single treatment. Unlike other targeted drugs, the combination of an antiangiogenic targeted drug and other drugs is not common. There is a contradiction between antiangiogenic targeted drugs and other cytotoxic drugs. In general, the antitumor effects of cytotoxic drugs depend on vascular transport. Apatinib and other antiangiogenic targeted drugs are used as a single treatment. Like other targeted drugs, the drug withdrawal can lead to rapid progression of the disease. In this study, 1 patient suffered rapid disease progression after drug withdrawal. Fortunately, his tumor was reduced again after the apatinib treatment was resumed. The drug dose is another concern. In this study, some children with advanced osteosarcoma received the apatinib at the dose of 250 mg daily, while adult patients received apatinib dose with 500 mg daily. Some patients had fluctuation of the use of medication due to the intolerable headache caused by drug-induced hypertension. In general, the symptoms were markedly relieved after reducing the dosage. The patients gradually returned to the normal dose after the symptoms became tolerable.

It is necessary to alert readers to be aware of the limitations of this study. Firstly, the number of patients is still small owing to the fact that osteosarcoma pulmonary metastases accepted apatinib treatments are not common. It has been reported that approximately 10% to 20% of patients present with metastatic disease at diagnosis.^[27,28]^ Three patients in this group present with metastatic disease at diagnosis while other patients present with metastatic disease after first-line chemotherapy. Both age distribution and peripheral location of pulmonary metastases in this study have good representativeness. Secondly, this is a retrospective study without control. It has been reported that the survival of patients with metastases at diagnosis or after a relapse remains poor and requires new therapeutic approaches.^[29]^ The results in this paper are written to share our experience but not deny other treatments. Thirdly, the antitumor drug mechanism of apatinib is not clear. There has been research conducted on the antitumor effect of apatinib on human osteosarcoma.^[30]^ The results showed that osteosarcoma patients with high levels of VEGFR2 have a poor prognosis and deactivation of the VEGFR2/STAT3/BCL2 signaling pathway leads to apatinib-induced growth inhibition of osteosarcoma. Whatever the complicated antitumor mechanism, the patients with osteosarcoma benefit from the apatinib treatment following failed first-line chemotherapy.

The results of this study indicate that apatinib is an effective treatment option for patients with osteosarcoma and pulmonary metastases after failed first-line chemotherapy. Thus, random controlled trials based on these data are warranted to further evaluate the apatinib activity.

## Acknowledgments

The authors thank everyone at our institution who helped in this study.

## Author contributions

**Conceptualization:** Kai Zheng, Xiuchun Yu.

**Data curation:** Kai Zheng, Ming Xu.

**Formal analysis:** Kai Zheng.

**Investigation:** Kai Zheng, Lei Wang, Xiuchun Yu.

**Methodology:** Kai Zheng, Xiuchun Yu.

**Project administration:** Ming Xu, Xiuchun Yu.

**Software:** Ming Xu.

**Supervision:** Ming Xu.

**Validation:** Lei Wang.

**Visualization:** Lei Wang.

**Writing – original draft:** Kai Zheng.

**Writing – review & editing:** Kai Zheng, Xiuchun Yu.

## References

[1] Siegel RL Miller KD Jemal A Cancer statistics, 2017. CA Cancer J Clin 2017;67:7–30. 28055103 10.3322/caac.21387

[2] Chen W Zheng R Baade PD Cancer statistics in China, 2015. CA Cancer J Clin 2016;66:115–32. 26808342 10.3322/caac.21338

[3] Goorin AM Shuster JJ Baker A Changing pattern of pulmonary metastases with adjuvant chemotherapy in patients with osteosarcoma: results from the multiinstitutional osteosarcoma study. J Clin Oncol 1991;9:600–5. 2066757 10.1200/JCO.1991.9.4.600

[4] Chi SN Conklin LS Qin J The patterns of relapse in osteosarcoma: the Memorial Sloan–Kettering experience. Pediatr Blood Cancer 2004;42:46–51. 14752794 10.1002/pbc.10420

[5] Briccoli A Rocca M Salone M High grade osteosarcoma of the extremities metastatic to the lung: long-term results in 323 patients treated combining surgery and chemotherapy, 1985–2005. Surg Oncol 2010;19:193–9. 19515554 10.1016/j.suronc.2009.05.002

[6] Bacci G Briccoli A Rocca M Neoadjuvant chemotherapy for osteosarcoma of the extremities with metastases at presentation: recent experience at the Rizzoli Institute in 57 patients treated with cisplatin, doxorubicin, and a high dose of methotrexate and ifosfamide. Ann Oncol 2003;14:1126–34. 12853357 10.1093/annonc/mdg286

[7] Cathomas R Rothermundt C Bode B RANK ligand blockade with denosumab in combination with sorafenib in chemorefractory osteosarcoma: a possible step forward. Oncology 2015;88:257–60. 25531914 10.1159/000369975

[8] Federman N Bernthal N Eilber FC The multidisciplinary management of osteosarcoma. Curr Treat Options Oncol 2009;10:82–93. 19238553 10.1007/s11864-009-0087-3

[9] Folkman J Antiangiogenesis in cancer therapy—endostatin and its mechanisms of action. Exp Cell Res 2006;312:594–607. 16376330 10.1016/j.yexcr.2005.11.015

[10] Hicklin DJ Ellis LM Role of the vascular endothelial growth factor pathway in tumor growth and angiogenesis. J Clin Oncol 2005;23:1011–27. 15585754 10.1200/JCO.2005.06.081

[11] Li J Qin S Xu J Randomized, Double-blind, placebo-controlled phase III trial of apatinib in patients with chemotherapy-refractory advanced or metastatic adenocarcinoma of the stomach or gastroesophageal junction. J Clin Oncol 2016;34:1448–54. 26884585 10.1200/JCO.2015.63.5995

[12] Zhang H Apatinib for molecular targeted therapy in tumor. Drug Des Devel Ther 2015;9:6075–81. 10.2147/DDDT.S97235 PMC4654530 26622168

[13] Li F Liao Z Zhao J Efficacy and safety of Apatinib in stage IV sarcomas: experience of a major sarcoma center in China. Oncotarget 2017;8:64471–80. 28969086 10.18632/oncotarget.16293 PMC5610018

[14] Zhou Y Zhang W Tang F A case report of apatinib in treating osteosarcoma with pulmonary metastases. Medicine (Baltimore) 2017;96:e6578. 28403086 10.1097/MD.0000000000006578 PMC5403083

[15] Zhou N Liu C Hou H Response to apatinib in chemotherapy-failed advanced spindle cell breast carcinoma. Oncotarget 2016;7:72373–9. 27738308 10.18632/oncotarget.12568 PMC5342168

[16] Li J Zhao X Chen L Safety and pharmacokinetics of novel selective vascular endothelial growth factor receptor-2 inhibitor YN968D1 in patients with advanced malignancies. BMC Cancer 2010;10:529. 20923544 10.1186/1471-2407-10-529 PMC2984425

[17] Zhang Y Han C Li J Efficacy and safety for apatinib treatment in advanced gastric cancer: a real world study. Sci Rep 2017;7:13208. 29038432 10.1038/s41598-017-13192-8 PMC5643341

[18] Yan P Sun ML Sun YP Effective apatinib treatment of pleomorphic liposarcoma: a case report. Medicine (Baltimore) 2017;96:e7771. 28816958 10.1097/MD.0000000000007771 PMC5571695

[19] Dong M Bi J Liu X Significant partial response of metastatic intra-abdominal and pelvic round cell liposarcoma to a small-molecule VEGFR-2 tyrosine kinase inhibitor apatinib: a case report. Medicine (Baltimore) 2016;95:e4368. 27495042 10.1097/MD.0000000000004368 PMC4979796

[20] Therasse P Arbuck SG Eisenhauer EA European Organization for Research and Treatment of Cancer, National Cancer Institute of the United States, National Cancer Institute of Canada. New guidelines to evaluate the response to treatment in solid tumors. J Natl Cancer Inst 2000;92:205–16. 10655437 10.1093/jnci/92.3.205

[21] Al-Abd AM Alamoudi AJ Abdel-Naim AB Anti-angiogenic agents for the treatment of solid tumors: potential pathways, therapy and current strategies—a review. J Adv Res 2017;8:591–605. 28808589 10.1016/j.jare.2017.06.006 PMC5544473

[22] Momeny M Sabourinejad Z Zarrinrad G Anti-tumour activity of tivozanib, a pan-inhibitor of VEGF receptors, in therapy-resistant ovarian carcinoma cells. Sci Rep 2017;7:45954. 28383032 10.1038/srep45954 PMC5382685

[23] Tian S Quan H Xie C YN968D1 is a novel and selective inhibitor of vascular endothelial growth factor receptor-2 tyrosine kinase with potent activity in vitro and in vivo. Cancer Sci 2011;102:1374–80. 21443688 10.1111/j.1349-7006.2011.01939.x PMC11158267

[24] Zhou Y Tang F Wang Y Advanced alveolar soft part sarcoma responds to apatinib. Oncotarget 2017;8:50314–22. 28679123 10.18632/oncotarget.18599 PMC5564851

[25] Grignani G Palmerini E Dileo P A phase II trial of sorafenib in relapsed and unresectable high-grade osteosarcoma after failure of standard multimodal therapy: an Italian Sarcoma Group study. Ann Oncol 2012;23:508–16. 21527590 10.1093/annonc/mdr151

[26] Grignani G Palmerini E Ferraresi V Sorafenib and everolimus for patients with unresectable high-grade osteosarcoma progressing after standard treatment: a non-randomised phase 2 clinical trial. Lancet Oncol 2015;16:98–107. 25498219 10.1016/S1470-2045(14)71136-2

[27] Kager L Zoubek A Pötschger U Primary metastatic osteosarcoma: presentation and outcome of patients treated on neoadjuvant Cooperative Osteosarcoma Study Group protocols. J Clin Oncol 2003;21:2011–8. 12743156 10.1200/JCO.2003.08.132

[28] Meyers PA Heller G Healey JH Osteogenic sarcoma with clinically detectable metastasis at initial presentation. J Clin Oncol 1993;11:449–53. 8445419 10.1200/JCO.1993.11.3.449

[29] Bielack SS Kempf-Bielack B Delling G Prognostic factors in high-grade osteosarcoma of the extremities or trunk: an analysis of 1,702 patients treated on neoadjuvant cooperative osteosarcoma study group protocols. J Clin Oncol 2002;20:776–90. 11821461 10.1200/JCO.2002.20.3.776

[30] Liu K Ren T Huang Y Apatinib promotes autophagy and apoptosis through VEGFR2/STAT3/BCL-2 signaling in osteosarcoma. Cell Death Dis 2017;8:e3015. 28837148 10.1038/cddis.2017.422 PMC5596600

